# In situ fixation of slipped capital femoral epiphysis with Steinmann pins: 67 patients followed for 2–16 years

**DOI:** 10.3109/17453674.2011.579520

**Published:** 2011-07-08

**Authors:** Trude G Lehmann, Ingvild Ø Engesæter, Lene B Laborie, Karen Rosendahl, Stein Atle Lie, Lars B Engesæter

**Affiliations:** ^1^Department of Orthopaedic Surgery, Haukeland University Hospital; ^2^Institute of Surgical Science, University of Bergen; ^3^Department of Radiology, Haukeland University Hospital; ^4^Uni Health, Uni Research, Bergen, Norway

## Abstract

**Background and purpose:**

Slipped capital femoral epiphysis (SCFE) is often treated by surgical fixation; however, no agreement exists regarding technique. We analyzed the outcome of in situ fixation with Steinmann pins.

**Patients and methods:**

All 67 subjects operated for slipped capital femoral epiphysis at Haukeland University Hospital during the period 1990–2007 were included. All were treated by in situ fixation with 2 or 3 parallel Steinmann pins (8 mm threads at the medial end). The follow-up evaluation consisted of clinical examination and hip radiographs. Radiographic outcome was based on measurements of slip progression, growth of the femoral neck, leg length discrepancy, and signs of avascular necrosis and chondrolysis.

**Results:**

67 subjects (41 males) were operated due to unilateral slips (n = 47) or bilateral slips (n = 20). Mean age at time of diagnosis was 13 (7.2–16) years. Mean age at follow-up was 19 (14–30) years, with a mean postoperative interval of 6.0 (2–16) years. The operated femoral neck was 9% longer at skeletal maturity than at surgery, indicating continued growth of the femoral neck. At skeletal maturity, 12 subjects had radiographic features suggestive of a previous asymptomatic slip of the contralateral hip. The total number of bilateral cases of SCFE was 32, i.e half of the children had bilateral SCFE. 3 subjects required additional surgery and mild avascular necrosis of the femoral head was seen in 1 patient. None had slip progression or chondrolysis.

**Interpretation:**

In situ pinning of SCFE with partly threaded Steinmann pins appears to be a feasible and safe method, with few complications. The technique allows further growth of the femoral neck.

Slipped capital femoral epiphysis (SCFE) is a disease of unknown etiology, but mechanical, biological and hereditary factors are likely to play a role (Barrios et al. 2005, Murray and Wilson 2008). The rationale for treatment of SCFE is to restore hip function, prevent further slip, and to reduce the risk of subsequent degenerative changes. Several surgical techniques have been recommended such as cannulated screws (Chen et al. 2009), hook-pins (Hansson 1982), specially constructed screws (Wensaas and Svenningsen 2005), and most recently surgical hip dislocation with subcapital correction osteotomy (Leunig et al. 2007). However, currently there is no evidence to support the superiority of one particular technique over another.

In situ fixation is advocated by most authors (Boyer et al. 1981, Carey et al. 1987, Givon and Bowen 1999) since peroperative reduction may increase the risk of avascular necrosis (Ordeberg et al. 1983, Carney et al. 1991, Lim et al. 2007). Physiodesis to prevent further growth—thus stabilizing the physis—is recommended by some authors (Carey et al. 1987, Aronsson and Karol 1996). Slip of the contralateral hip is reported in more than half of the cases (Hägglund et al. 1988, Castro et al. 2000) and controversies exist regarding prophylactic fixation of the contralateral hip. According to Jerre et al. (1994), more than two-thirds of the contralateral slips are asymptomatic and are therefore only detected at close follow-ups including hip radiographs at short intervals. Immediate prophylactic fixation of the contralateral hip has been advocated by several authors (Hägglund et al. 1988, Schultz et al. 2002, Krauspe et al. 2004).

In this paper, we present clinical and radiographic results of a novel, simple technique for in situ fixation of the femoral head with partially threaded Steinmann pins to enable further growth of the femoral neck.

## Patients and methods

All 67 subjects operated for SCFE at Haukeland University Hospital (Norway) during the period 1990–2007 were approached by mail in 2008 and were invited to participate in a follow-up including a clinical and radiographic assessment. Data on age at diagnosis and sex, and clinical data (duration and type of preoperative symptoms, technique, and duration of surgery) were collected from the medical records. The slips were classified according to Herring (2008): acute slip (onset of symptoms within 3 weeks of the diagnosis), acute-on-chronic slip (symptoms for more than 3 weeks with an acute deterioration over the most recent 3 weeks), chronic slip (symptoms for more than 3 weeks), and pre-slip (pain and clinical findings in the contralateral hip without any radiographic evidence of SCFE). A pelvic radiograph (frog-leg view) at the time of diagnosis was used to classify the degree of slip into mild, moderate, or severe based on measurements of the lateral epiphyseal shaft angle (Southwick 1967). The slip was considered mild if the angle was less than 30°, moderate if the angle was 30–50°, and severe when the angle was more than 50° (Boyer et al. 1981, Carney et al. 1991). In cases where there were missing radiographs (n = 21), data from the medical records or from the radiographic report were used to classify the degree of slip. We did not have information on the stability of the slip in all subjects.

**Figure 1.**
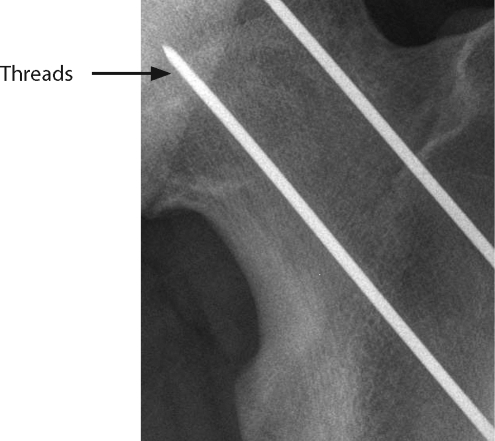
Steinmann pin with threads in the medial 8-mm tip.

**Figure 2.**
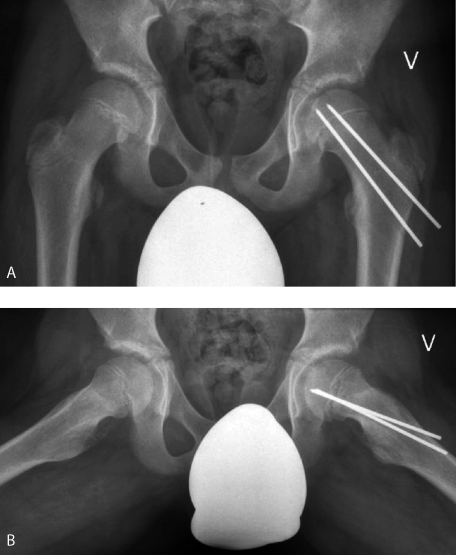
Postoperative radiographs of a 14-year-old boy after percutaneous pinning of SCFE. A. supine AP view. B. Frog-leg view.

The surgical procedure was performed with the child supine on a traction table. A uniplane or biplane image intensifier was used. The surgeon was responsible for placing the affected leg in traction on the operating table, to avoid forceful reduction. In children with acute or acute-on-chronic SCFE, very gentle repositioning was allowed (careful internal rotation on a flexed hip). The surgeon on call performed the operation, i.e. 42 surgeons performed between 1 and 13 operations each during the study period. A percutaneous fixation technique was used, with a 2–3-cm skin incision. 2 or 3 parallel Steinmann pins (diameter 2.3 mm) with threads in the 8-mm medial end (Figure 1) (Smith and Nephew, Menphis, TN) were drilled in under fluoroscopic guidance. To ease the placement and to protect the soft tissue, a drill guide was used. During the first 10-year period, we used 3 pins (n = 39), while 2 were used for the rest of the period (n = 45). 3 children had 4 pins inserted at the start of the study. Pins were cut 1–2 cm from the lateral femoral cortex (Figure 2). Postoperatively, the child was mobilized with crutches and partially weight-bearing for 4–6 weeks; thereafter, there were no restrictions. None of the children had prophylactic pinning of the contralateral hip. Subjects were followed annually at the outpatient clinic, until closure of the proximal femoral physis. The pins were then removed under general anesthesia.

### Outcome

Clinical and radiographic findings after physeal closure were used to assess long-term outcome (n = 60 subjects). 4 subjects (5 hips) did not attend the final follow-up (after 1 reminder) and 3 subjects (5 hips) had not yet reached skeletal maturity with closure of the proximal femoral growth plate. These 7 subjects were excluded from the analysis of long-term outcome.

**Figure 3.**
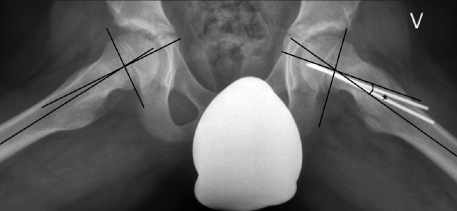
Measurement of the lateral epiphyseal shaft angles on a frog-leg view in a 14-year-old boy (the left angle is marked with an asterisk). The angle is measured in the following way: 90° minus the measured angle between a mid-diaphyseal line and a line through the anterior and posterior aspects of the physis. Normal angle on the right side (4°) and an angle of 23° on the left side.

**Figure 4.**
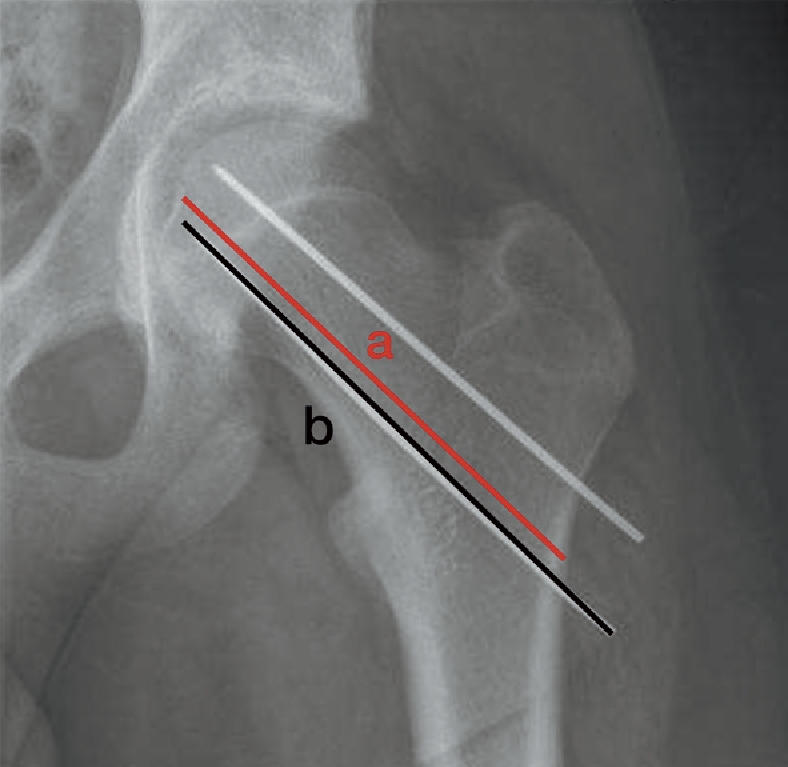
Method of calculating the ratio between lengths of the femoral neck and pin. a: length of femoral neck; b: length of Steinmann pin. Ratio: a/b.

The radiographic examination at the final follow-up included 2 supine views (1 anteroposterior (AP) and 1 frog-leg view). Radiographic outcomes were slip progression of more than 10° as assessed by Southwick's lateral epiphyseal-shaft angle (Southwick 1967) (Figure 3), signs of avascular necrosis (Kalamchi and MacEwen 1980), leg length discrepancy as assessed by differences in articulotrochanteric distance (ATD) (measured from the superior margins of the greater trochanter to the superior margins of the femoral head), longitudinal growth of the femoral neck, and whether there was evidence of a chondrolysis. Longitudinal growth of the femoral neck was estimated by constructing a ratio between the length of the femoral neck to the length of the Steinmann pin, as measured on the AP pelvic radiograph (Figure 4). The ratio was calculated on the first postoperative radiograph and the latest radiograph before pin removal. The mean difference in ratio was calculated. Chondrolysis was defined as more than 50% reduction of minimal joint space compared to the contralateral side (Loder et al. 2000). Slip progression was defined as an increased lateral epiphyseal shaft angle by more than 10° from surgery to final follow-up at skeletal maturity (Carney et al. 2003). In accordance with Jerre, we considered a lateral epiphyseal-shaft angle above 13° in the asymptomatic, contralateral hip to be diagnostic of a silent slip (Loder et al. 1993, Jerre et al. 1994).

Clinical long-term outcomes were based on clinical assessment of bilateral hip motions for subjects operated unilaterally and without signs of an asymptomatic slip at follow-up (n = 31). To increase the accuracy of the radiographic assessment, all radiographs were re-measured by one of the authors (TL). Repeatability of the measurements was evaluated by re-reading all images (masked regarding other data) after 6 months.

### Ethics

The procedures followed were in accordance with the ethical standards of the Regional Ethical Committee for Medical and Health Research. Written informed consent was obtained from all participants.

### Statistics

Femoral neck-pin ratio postoperatively and at skeletal maturity was compared using paired sample t-test. Association between severity of slip and duration of symptoms was analyzed by one-way ANOVA. Intra-observer variation for continuous variables was assessed by estimating the mean differences and their standard deviations (SDs). We then calculated the mean differences × 1.96 SD, expecting 95% of the differences between measurements to lie between these limits (repeatability coefficient as suggested by Bland and Altman (2003). All p-values < 0.05 were considered statistically significant. The analyses were performed using SPSS for Windows version 17.0. Clustered observations were controlled for using the statistical program gllamm in Stata software version 11.

## Results

67 subjects (41 males) with 87 slips were operated during the study period of 18 years (1990–2007) (Table). Mean age at diagnosis was 13 (7.2–16) years: 13 years for boys and 12 years for girls. 47 subjects had unilateral involvement (33 left hips) and 20 had bilateral slips.

**Table:** Severity and type of slip (given for each hip) and symptoms at the time of diagnosis (given for each patient) in 67 subjects with 87 slips

	Males	Females	Total
Severity of slip (87 hips)			
mild	32 (37%)	11 (13%)	43 (49%)
moderate	16 (18%)	12 (14%)	28 (32%)
severe	7 (8%)	7 (8%)	14 (16%)
not classified	0	2 (2%)	2 (2%)
Type of slip (87 hips)			
acute slip	6 (7%)	6 (7%)	12 (14%)
chronic slip	36 (41%)	17 (20%)	53 (61%)
acute-on-chronic slip	8 (9%)	6 (7%)	14 (16%)
pre-slip	5 (6%)	3 (3%)	8 (9%)
Duration of symptoms (months)			
mean (SD) (67 subjects)	5.2 (6.3)	5.6 (6.6)	5.3 (6.4)
mild slip	3.5 (5.2)	2.3 (2.3)	3.2 (4.7)
moderate slip	8.3 (8.3)	6.4 (5.5)	7.4 (7.2)
severe slip	5.8 (1.5)	10.6 (9.7)	8.4 (7.4)
Symptoms (87 hips)			
limp	40 (73%)	27 (84%)	67 (77%)
pain	55 (100%)	31 (97%)	86 (99%)
–hip/thigh	40 (73%)	25 (78%)	65 (75%)
– thigh/knee	7 (13%)	3 (9%)	10 (11%)
– knee	3 (5%)	2 (6%)	5 (6%)
– hip/thigh/knee	5 (9%)	1 (3%)	6 (7%)

Of the 20 individuals with bilateral slips, only 5 (2 males) presented with bilateral symptoms and had immediate bilateral surgery. For the 15 children who suffered sequential slips, mean age at diagnosis of the initial slip (8 mild, 7 moderate, 3 severe, and 2 unclassified) was 13 (11–16) years, 11 (11–12) years for girls and 13 (12–16) years for boys. The contralateral slip was operated on average 9 (1–36) months after the initial operation.

Children with moderate or severe slips had longer duration of symptoms than children with mild slips (p = 0.004). Symptom duration was similar in children with moderate slips and in those with severe slips. 4 children had additional disease associated with SCFE: 1 had trisomy for chromosome 21 (10 years of age), 2 had hypopituitarism (11 and 16 years old), and 1 received growth hormone medication due to hormonal deficiency (12 years old).

### Surgery

45 of the hips were treated with 2 Steinmann pins, 39 hips had 3 pins inserted, and in 3 hips 4 pins were used. The mean duration of the operation was 40 (10–105) min, 36 min in those having 2 pins inserted including 5 children with bilateral procedures (with total operation time divided by 2 for each side). There were acute surgical complications in 10 hips; in 3, the postoperative radiographs showed that the pins were penetrating the joint (all of these were replaced after 1–2 days) and in 5 the pins protruded too far into the soft tissues laterally (these were shortened within 24 h). 2 patients received antibiotics for a superficial wound infection. None of these 10 patients suffered long-term sequelae. Later, additional surgery was required in 3 children, of whom 1 had a femur lengthening osteotomy due to a leg length discrepancy of 2.5 cm, 1 needed a re-fixation of the femoral head due to displaced pins, and 1 had a fracture to the femur 2 weeks after pin removal.

The mean time from operation until removal of the pins was 3.3 (1.0–7.1) years. Mean duration of pin removal was 47 (10–146) min (the one subject with operation time of 146 min had removal of pins done in same session as lengthening osteotomy with intramedullary nailing). All subjects who were operated on for bilateral SCFE had their pins removed in 1 session. For these, operation time was calculated as the total time divided by 2. There were no pin fractures during removal. At follow-up, the pins had been removed in 74 of the 87 hips. In 4 of the remaining hips, the pins were entirely embedded in bone and were thus not removed, while 4 subjects declined. 3 subjects (5 hips) had not reached skeletal maturity at the last follow-up.

### Long-term outcome

The 60 subjects with long-term follow-up (see above) had a mean age at follow-up of 19 (14–30) years, with a mean follow-up time of 6.0 (2–16) years after surgery. 1 person had radiographic evidence of mild avascular necrosis. No cases of chondrolysis or slip progression were seen.

Mean femoral neck-pin ratio postoperatively was 0.92 (0.73–1.0) and at skeletal maturity it was 1.0 (0.80–1.2). Mean difference in femoral neck-pin ratio at skeletal maturity was 0.08 (–0.08 to 0.31) compared to first postoperative measurement, and the ratio increased in all but 1 hip. The femoral neck had a 9% increase in length at skeletal maturity compared to the length postoperatively (p < 0.001).

For 31 subjects who had unilateral surgery without any signs or suggestions of contralateral involvement, movement in the operated hip was compared to that in the normal hip. A mean reduction of 5º (SD = 11) in internal rotation and a mean increase of 9º (SD = 9) in external rotation was found for the operated hip. These were not statistically significant, however. The mean difference in ATD between the operated hip and the contralateral hip for subjects operated unilaterally was 7.3 (0–17) mm.

At follow-up, 12 subjects (6 males) had radiographic findings suggestive of a contralateral slip (11 mild, 1 moderate) which had not been diagnosed previously. The mean age at diagnosis of the initial slip for these subjects was 12 (7.2–15) years. The mean Southwick's angle for the asymptomatic slip was 19° (13–33). Thus, 32 of the 67 subjects (20 males) had bilateral slips at follow-up.

Intraobserver variation for Southwick's angle and ATD was acceptable, with the following mean differences (SD) and their repeatability coefficients (n = 64). Southwick's angle, right hip: –0.5º (SD 2.5), –5.4º to 4.4º; and left hip: –0.03º (SD 3.3), –6.5º to 6.5º. ATD, right hip: 0.2 mm (SD 1.1), –2.0 mm to 2.4 mm; and left hip: 0.2 mm (SD 1.5), –2.7 mm to 3.1 mm. Analyses taking the few clustered observations into account did not alter any of the findings in this study.

## Discussion

This study indicates that in situ pinning of slipped capital femoral epiphysis with partly threaded Steinmann pins is a feasible and safe technique with few peroperative and postoperative complications, and with good clinical and radiographic long-term outcome. The technique enables further growth of the femoral neck, with an acceptable leg length discrepancy at skeletal maturity. None of the operated hips had a slip progression of more than 10°.

Before introducing this new surgical technique for stabilization of the epiphysis in SCFE, we showed in a laboratory setting that the mechanical strength of Steinmann pins was sufficient for fixation of human femoral neck osteotomies and accordingly also for SCFE (Rynning et al. 1990). We initially used 3 Steinmann pins, but later reduced the number to 2.

Slip progression after stabilization with a single screw has been reported by several authors (Carney et al. 1991, Aronson and Carlson 1992, Denton 1993). Carney et al. (2003) found that 20% suffered a slip progression of 10° or more when operated with a single cannulated screw. The idea that double screw fixation is more likely to provide torsional stability in non-reduced slips than a single screw has been verified in artificially created slips in bovine femurs (Segal et al. 2006).

Others have used multiple Kirchner wires to fixate the femoral head. In a study of 29 patients, a repeat transfixation was judged to be necessary in 7 of the cases as the wires lost contact with the femoral head during growth (Seller et al. 2006). We believe that our favorable results may be due in part to the threads at the end of the pins, securing sufficient anchorage within the femoral head during the residual growth.

Avascular necrosis (AVN) of the femoral head is a severe surgical complication. Carey et al. (1987) reviewed 60 patients operated with threaded pin fixation. At follow-up of between 4 and 13 years, 8 patients had findings consistent with AVN. Carney et al. (1991) reported on 155 operated hips with a mean follow-up of 41 years. AVN was diagnosed in 12% of the subjects, and was more frequent in those with severe slips. They also found a positive association between AVN and penetration of a pin into the joint. We found 1 mild AVN, with partial involvement of the femoral head, in a boy with a severe slip after having had symptoms for 3.5 months. He had 3 pins inserted, and later suffered a leg length discrepancy of 2.5 cm, which was subsequently treated with a leg lengthening procedure. AVN was not seen in any of the 3 subjects who had pin penetration to the joint.

The most serious complication seen was a subtrochanteric femoral fracture 2 weeks after pin removal, most likely caused by extensive bone chiselling. We no longer remove pins that are embedded in bone. Accordingly, in 4 subjects the pins were not removed.

The rationale for treatment in SCFE is to prevent further epiphyseal slip. Some authors advocate that this is best achieved through artificial fusion of the proximal growth plate. However, this may lead to leg length discrepancy, more so in younger subjects, and also to overgrowth of the greater trochanter (Howorth 1966). Such overgrowth may again lead to impingement and reduced abduction forces, with limping. It has been argued that prophylactic pinning of the contralateral hip may reduce the risk of leg length discrepancy (Castro et al. 2000, Riad et al. 2007). To our knowledge, none of the studies favoring surgical closure of the growth plate have examined leg length discrepancy at skeletal maturity.

Remodeling after SCFE results from bone deposition anteromedially and absorption posterolaterally. Several authors claim that remodeling also results from reduced Southwick's angle (Bellemans et al. 1996). In theory, this reduced angle may result from further asymmetric growth of the femoral neck. Our results indicate that stabilization of the epiphysis from further slip is possible without stopping the longitudinal growth of the femoral neck. This may lead to better biomechanics, improved remodeling, and reduced leg length discrepancy. When using ATD as a measure of leg length discrepancy, it should be kept in mind that the ATD is dependent on the degree of initial slip, the remodeling, and the growth of the femoral neck.

Bilateral involvement was seen in half of the subjects, one third of which were undiagnosed until the subjects were adults. In a long-term follow-up of 260 patients from 1988, Hägglund et al. found that 61% had bilateral slips at skeletal maturity, 40% of which remained undiagnosed until the long-term follow-up. In another study involving 224 children, Loder et al. (1993) reported that 37% had bilateral slips. In a retrospective study of 100 patients, Jerre et al. (1996) found bilateral slip in 59% after 32 years observation. Around two-thirds of these were asymptomatic, and 18% were first diagnosed after skeletal maturity. As demonstrated by Bellemans et al. (1996), remodeling after SCFE also results from reduced Southwicks angle. This remodeling may result in an underestimation of bilateral slips. Small slips may have remodeled, resulting in a head-shaft angle that is found to be normal at follow-up (Clarke and Harrison 1986).

In a report published by Hägglund in 1996, 25% of patients with an undiagnosed slip had coxarthrosis before the age of 50, and during recent years it has been discussed that even silent slips could be the cause of femoroacetabular impingement. This shows that even the minor slips may give problems later in life, and that preventing a silent slip may be important. Based on the information in the literature and on our results, we have now changed the clinical routine in our department to prophylactic pinning of the contralateral hip in children presenting with a unilateral slip.

## References

[CIT0001] Aronson DD, Carlson WE (1992). Slipped capital femoral epiphysis. A prospective study of fixation with a single screw. J Bone Joint Surg (Am).

[CIT0002] Aronsson DD, Karol LA (1996). Stable Slipped Capital Femoral Epiphysis: Evaluation and Management. J Am Acad Orthop Surg.

[CIT0003] Barrios C, Blasco MA, Blasco MC, Gasco J (2005). Posterior sloping angle of the capital femoral physis: a predictor of bilaterality in slipped capital femoral epiphysis. J Pediatr Orthop.

[CIT0004] Bellemans J, Fabry G, Molenaers G, Lammens J, Moens P (1996). Slipped capital femoral epiphysis: a long-term follow-up, with special emphasis on the capacities for remodeling. J Pediatr Orthop B.

[CIT0005] Bland JM, Altman DG (2003). Applying the right statistics: analyses of measurement studies. Ultrasound Obstet Gynecol.

[CIT0006] Boyer DW, Mickelson MR, Ponseti IV (1981). Slipped capital femoral epiphysis—long-term follow-up-study of 121 patients. J Bone Joint Surg (Am).

[CIT0007] Carey RP, Moran PL, Cole WG (1987). The place of threaded pin fixation in the treatment of slipped upper femoral epiphysis. Clin Orthop.

[CIT0008] Carney BT, Weinstein SL, Noble J (1991). Long-Term Follow-Up of Slipped Capital Femoral Epiphysis. J Bone Joint Surg (Am).

[CIT0009] Carney BT, Birnbaum P, Minter C (2003). Slip progression after in situ single screw fixation for stable slipped capital femoral epiphysis. J Pediatr Orthop.

[CIT0010] Castro FP, Bennett JT, Doulens K (2000). Epidemiological perspective on prophylactic pinning in patients with unilateral slipped capital femoral epiphysis. J Pediatr Orthop.

[CIT0011] Chen RC, Schoenecker PL, Dobbs MB, Luhmann SJ, Szymanski DA, Gordon JE (2009). Urgent reduction, fixation, and arthrotomy for unstable slipped capital femoral epiphysis. J Pediatr Orthop.

[CIT0012] Clarke NM, Harrison MH (1986). Slipped upper femoral epiphysis. A potential for spontaneous recovery. J Bone Joint Surg (Br).

[CIT0013] Denton JR (1993). Fixation with a single screw for slipped capital femoral epiphysis. J Bone Joint Surg (Am).

[CIT0014] Givon U, Bowen JR (1999). Chronic slipped capital femoral epiphysis: treatment by pinning in situ. J Pediatr Orthop B.

[CIT0015] Hägglund G (1996). The contralateral hip in slipped capital femoral epiphysis. J Pediatr Orthop B.

[CIT0016] Hägglund G, Hansson LI, Ordeberg G, Sandstrøm S (1988). Bilaterality in Slipped upper Femoral Epiphysis. J Bone Joint Surg (Br).

[CIT0017] Hansson LI (1982). Osteosynthesis with the hook-pin in slipped capital femoral epiphysis. Acta Orthop Scand.

[CIT0018] Herring JA (2008). Tachdjian's pediatric orthopaedics.

[CIT0019] Howorth B (1966). The bone-pegging operation for slipping of the capital femoral epiphysis. Clin Orthop.

[CIT0020] Jerre R, Billing L, Hansson G, Wallin J (1994). The contralateral hip in patients primarily treated for unilateral slipped upper femoral epiphisis- Long-term follow-up of 61 hips. J Bone Joint Surg (Br).

[CIT0021] Jerre R, Billing L, Hansson G, Karlsson J, Wallin J (1996). Bilaterality in slipped capital femoral epiphysis: Importance of a reliable radiographic method. J Pediatr Orthop B.

[CIT0022] Kalamchi A, MacEwen GD (1980). Avascular necrosis following treatment of congenital dislocation of the hip. J Bone Joint Surg (Am).

[CIT0023] Krauspe R, Seller K, Westhoff B (2004). Slipped capital femoral epiphysis. Z Orthop Ihre Grenzgeb.

[CIT0024] Leunig M, Slongo T, Kleinschmidt M, Ganz R (2007). Subcapital correction osteotomy in slipped capital femoral epiphysis by means of surgical hip dislocation. Oper Orthop Traumatol.

[CIT0025] Lim YJ, Lam KS, Lim KB, Mahadev A, Lee EH (2007). Management outcome and the role of manipulation in slipped capital femoral epiphysis. J Orthop Surg (Hong Kong ).

[CIT0026] Loder RT, Aronson DD, Greenfield ML (1993). The Epidemiology of Bilateral Slipped Capital Femoral Epiphysis - A Study of Children in Michigan. J Bone Joint Surg (Am).

[CIT0027] Loder RT, Aronsson DD, Dobbs MB, Weinstein SL (2000). Slipped capital femoral epiphysis. J Bone Joint Surg (Am).

[CIT0028] Murray AW, Wilson NI (2008). Changing incidence of slipped capital femoral epiphysis: A relationship with obesity?. J Bone Joint Surg (Br).

[CIT0029] Ordeberg G, Hansson LI, Sandstrom S (1983). Long-term results in slipped capital femoral epiphysis with different primary-treatment. Acta Orthop Scand.

[CIT0030] Riad J, Bajelididze G, Gabos PG (2007). Bilateral slipped capital femoral epiphysis—predictive factors for contralateral slip. J Pediatr Orthop.

[CIT0031] Rynning SE, Engesæter LB, Mølster AO, Gjerdet NR (1990). Stability of osthesyntheses used in femoral neck fractures: An experimental study comparing the strength of five different osteosyntheses. Acta Orthop Scand (Suppl 239).

[CIT0032] Schultz WR, Weinstein JN, Weinstein SL, Smith BG (2002). Prophylactic pinning of the contralateral hip in slipped capital femoral epiphysis: evaluation of long-term outcome for the contralateral hip with use of decision analysis. J Bone Joint Surg (Am).

[CIT0033] Segal LS, Jacobson JA, Saunders MM (2006). Biomechanical analysis of in situ single versus double screw fixation in a nonreduced slipped capital femoral epiphysis model. J Pediatr Orthop.

[CIT0034] Seller K, Wild A, Westhoff B, Raab P, Krauspe R (2006). Clinical outcome after transfixation of the epiphysis with Kirschner wires in unstable slipped capital femoral epiphysis. Int Orthop.

[CIT0035] Southwick WO (1967). Osteotomy through the lesser trochanter for slipped capital femoral epiphysis. J Bone Joint Surg (Am).

[CIT0036] Wensaas A, Svenningsen S (2005). Behandling av epiphysiolysys capitis femoris med spesialkonstruert collumskrue. Tidsskr Nor Laegeforen.

